# CaMKII and PKA-dependent phosphorylation co-regulate nuclear localization of HDAC4 in adult cardiomyocytes

**DOI:** 10.1007/s00395-021-00850-2

**Published:** 2021-02-15

**Authors:** Kathryn G. Helmstadter, Senka Ljubojevic-Holzer, Brent M. Wood, Khanha D. Taheri, Simon Sedej, Jeffrey R. Erickson, Julie Bossuyt, Donald M. Bers

**Affiliations:** 1 Department of Pharmacology, University of California, Genome Building Rm 3513, Davis, CA 95616-8636 USA; 2 Department of Cardiology, Medical University of Graz, Graz, Austria; 3 BioTechMed-Graz, Graz, Austria; 4 Department of Physiology and HeartOtago, University of Otago, Dunedin, New Zealand

**Keywords:** Histone deacetylase 4 (HDAC4), Calcium-calmodulin-dependent protein kinase (CaMKII), Protein kinase A (PKA), Ventricular remodeling, Cardiac hypertrophy

## Abstract

Nuclear histone deacetylase 4 (HDAC4) represses MEF2-mediated transcription, implicated in the development of heart failure. CaMKII-dependent phosphorylation drives nucleus-to-cytoplasm HDAC4 shuttling, but protein kinase A (PKA) is also linked to HDAC4 translocation. However, the interplay of CaMKII and PKA in regulating adult cardiomyocyte HDAC4 translocation is unclear. Here we sought to determine the interplay of PKA- and CaMKII-dependent HDAC4 phosphorylation and translocation in adult mouse, rabbit and human ventricular myocytes. Confocal imaging and protein analyses revealed that inhibition of CaMKII—but not PKA, PKC or PKD—raised nucleo-to-cytoplasmic HDAC4 fluorescence ratio (F_Nuc_/F_Cyto_) by ~ 50%, indicating baseline CaMKII activity that limits HDAC4 nuclear localization. Further CaMKII activation (via increased extracellular [Ca^2+^], high pacing frequencies, angiotensin II or overexpression of CaM or CaMKIIδC) led to significant HDAC4 nuclear export. In contrast, PKA activation by isoproterenol or forskolin drove HDAC4 into the nucleus (raising F_Nuc_/F_Cyto_ by > 60%). These PKA-mediated effects were abolished in cells pretreated with PKA inhibitors and in cells expressing mutant HDAC4 in S265/266A mutant. In physiological conditions where both kinases are active, PKA-dependent nuclear accumulation of HDAC4 was predominant in the very early response, while CaMKII-dependent HDAC4 export prevailed upon prolonged stimuli. This orchestrated co-regulation was shifted in failing cardiomyocytes, where CaMKII-dependent effects predominated over PKA-dependent response. Importantly, human cardiomyocytes showed similar CaMKII- and PKA-dependent HDAC4 shifts. Collectively, CaMKII limits nuclear localization of HDAC4, while PKA favors HDAC4 nuclear retention and S265/266 is essential for PKA-mediated regulation. These pathways thus compete in HDAC4 nuclear localization and transcriptional regulation in cardiac signaling.

**Supplementary Information:**

The online version contains supplementary material available at 10.1007/s00395-021-00850-2.

## Introduction

The heart undergoes many changes as it progresses towards failure, one of which is the reactivation of the fetal gene program, resulting in the upregulation of β-MHC, ANP, and BNP gene expression [14]. Histone deacetylase 4 (HDAC4) is hypothesized to play a key role in the progression to pathological hypertrophy during structural heart disease [27] because of its ability to repress myocyte enhancing factor 2 (MEF2)-mediated transcription [29, 63]. MEF2 transcription factors bind to the gene regulatory elements of DNA in muscle-specific genes, influencing myogenesis and, e.g., expression of α- and β-MHC genes [37, 49].

HDAC4 localization critically determines its function in myocytes, as HDAC4 acts as potent allosteric corepressors of MEF2 transcriptional activity in the nucleus [27] and aids in regulating the contractile activity of myofilaments in the cytosol [28]. Phosphorylation of three serine residues (246, 467, and 632) play a key role in modulating HDAC4 nuclear export because these phosphoserines are recognized by the chaperone protein 14-3-3 [26, 64]. 14-3-3 binding prevents importin-α from binding to the HDAC4 nuclear localization sequence (NLS) [26, 50, 65] and exposes the nuclear export sequence (NES) at the C-terminus [47, 65], resulting in nuclear export and cytosolic retention of HDAC4. The NES is recognized by the exportin, CRM-1, which is known to participate in HDAC4 nuclear export [65].

Several kinases can phosphorylate S246, S467, or S632 in cell lines and in neonatal myocytes, including CaMKII [2, 4, 39], PKD [13, 46], and Mark-2 [13]. Notably, HDAC4 contains a unique CaMKII-docking site, R601, which is required for CaMKII to exhibit its predominant role as the key HDAC4 kinase in hypertrophic signaling [4]. In COS cells PKA was shown to bind to HDAC4 and cause cleavage of an N-terminal portion of HDAC4 containing the MEF2 binding domain (but none of the phosphorylation sites) and this fragment inhibited MEF2 function [5]. Despite extensive study of HDAC4 in generic cell lines and some studies in neonatal ventricular myocytes, little is known about control of HDAC4 subcellular distribution in adult ventricular myocytes. In addition, in various cell types and in vitro settings, HDAC4 appears to be substrate also for PKA [20, 40], however, no evidence of PKA-dependent phosphorylation effects has been reported for HDAC4 in adult cardiomyocytes.

Because HDAC4 phosphorylation by specific cellular kinases directly couples many stress signals to transcriptional regulation in the nucleus, detailed phenotyping of upstream kinases that regulate HDAC4 localization in the heart is crucial for molecular understanding of cardiac stress responses and moving toward translational cardiac therapies involving class II HDACs. Here, using adult ventricular myocytes from mouse, rabbit and human hearts, we demonstrate that the baseline nucleo-to-cytoplasmic ratio (F_Nuc_/F_Cyto_) of HDAC4 levels is limited already by CaMKII at rest and further nuclear export occurs via CaMKII activation. However, β-adrenergic stimulation, via the activity of cAMP-dependent protein kinase (PKA) results in the nuclear retention/import of HDAC4 and S265/266 is essential for PKA-mediated regulation. This novel finding sheds light on crosstalk between CaMKII and PKA-dependent signaling, relevant to β-blocker effects in hypertrophy and heart failure.

## Materials and methods

### Ethical consideration

The data supporting findings of this study are available from the corresponding author upon reasonable request. All procedures involving animals adhered to the NIH Guide for the Care and Use of Laboratory Animals (UC Davis) or the Federal Act on the Protection of Animals (Medical University of Graz) and were approved by the Institutional Animal Care and Use Committee. Human hearts (from patients and organ donors whose hearts could not be used for transplantation) were acquired via the California Transplant Donor Network (UC Davis) or the collaboration with the Division of Cardiac Surgery (Medical University of Graz). Human sample use was approved by the Ethical Committee of the University of California, San Francisco and Davis and the Medical University of Graz and all experimental procedures were carried out in accordance with the *Declaration of Helsinki*.

### Transgenic mice

Transgenic mice expressing CaMKIIδC in the heart (TG) and knockout mice lacking CaMKIIδ (KO) were generated as described previously [38, 71]. Black Swiss C57BL/6 mice of mixed genders and 10–12 weeks of age were used in the experiments, and age-matched WT littermates served as controls.

### Rabbit model of heart failure

Heart failure was induced in New Zealand White rabbits by combined aortic insufficiency and abdominal aortic stenosis as previously described [35, 54]. Heart failure progression was assessed by 2D echocardiography and rabbits were studied ~ 12 months later, when end-systolic dimension exceeded 12 mm [54].

### Myocyte isolation and culture

Rabbit and mouse ventricular myocytes were isolated as previously described [6, 42]. Freshly isolated cells were plated on laminin-coated glass coverslips or onto glass-bottomed 4 or 8-well chambers (Nunc™, Thermo Fisher Scientific, USA). Rabbit ventricular myocytes were cultured for 24–40 h at 37 °C in PC-1 media (Lonza, USA) with 1.8 mM Ca^2+^. Cardiomyocytes from human ventricular tissue were isolated using previously established protocol [19].

### Adenoviral infection

Isolated adult rabbit myocytes were infected an hour after plating with replication-deficient adenovirus expressing HDAC4-GFP, HDAC4-S265/266A-GFP or HDAC4-R601F-GFP, with GFP on the C-terminal of HDAC4 at an MOI of 100. Where indicated, cells were alternatively infected with replication-deficient adenovirus expressing HDAC5-GFP at an MOI of 10, calmodulin (CaM) at an MOI of 100, or CaMKIIδC at an MOI of 100 and maintained in culture overnight.

### Reagents

KN93, H-89, protein kinase inhibitor (PKI), bisindolylmaleimide I (Bis I), forskolin, isoproterenol (Iso), angiotensin II (Ang II), staurosporine, okadaic acid (OA) and leptomycin B (LMB) were all from Calbiochem (USA).

### Immunoprecipitation (IP)

We followed commercial instructions (Thermo Fisher Scientific, USA) with minor changes for the IP procedure. Our IP Buffer was supplemented with protease inhibitor and 1.5% NP-40. We used a 14-3-3 antibody (#sc-25276, Santa Cruz Biotechnology, USA) to pull down HDAC4-GFP and Protein G Plus UltraLink Resin (#53128, Thermo Fischer Scientific, USA) to bind the antigen–antibody complex. Samples were evaluated by SDS-PAGE and immunoblotting was done using a GFP-specific antibody (#ab290-50, Abcam, USA) at 1:10,000.

### Immunocytochemistry (ICC)

Isolated adult rabbit cardiomyocytes were plated on laminin-coated 8-well chambers (Nunc™, Thermo Fisher Scientific, USA) and cultured overnight at 37 °C in PC-1 media (Lonza, USA) with 1.8 mM Ca^2+^ to mimic conditions used in cells infected with adenovirus. Unless otherwise indicated, we used following concentrations of pharmacological inhibitors or agonists (in µM): 1 staurosporine, 1 KN93, 2 H-89, 20 PKI, 10 Bis I, 10 Gö6976, 0.1 or 1 OA, 0.1 Ang II, 1 Iso and 10 forskolin. Cultured cardiomyocytes were fixed with 4% PFA for 10 min. Fixed cells were rinsed with 1xPBS + 100 mM glycine, then 1xPBS. Cells were permeabilized with 0.2% Triton (Sigma, USA) dissolved in “Antibody Dilution Buffer” (1xPBS + 3% Fraction V BSA, 7.5% goat serum [KPL, #71-00-27]) for 10 min. Cells were rinsed with 1xPBS + 1%BSA and then blocked with a 1:1 mixture of 10% goat serum and 10% BSA for 1–2 h at room temperature (25 °C). Cells were rinsed with 1xPBS + 1%BSA and then incubated overnight at 4 °C in primary antibody against HDAC4 (#sc-11418, Santa Cruz Biotechnology, USA) at 1:150 dilution in antibody dilution buffer. The following day, cells were rinsed with 1xPBS + 1%BSA several times and then secondary antibody was added (Alexa fluor 488, #A-11008, Invitrogen, USA) at 1:50 dilution in Antibody Dilution Buffer and incubated overnight at 4 °C, protected from light. The following day, cells were rinsed with 1xPBS + 1%BSA, then with PBS two times. Confocal imaging of labeled cells was performed immediately afterwards using an inverted microscope equipped with a Plan Neofluar 40x/1.3N.A. oil-immersion objective and a Zeiss LSM5 laser scanning microscope (Zeiss, Germany). Excitation and emission wavelengths were 488 nm and > 515 nm, respectively. Imaging configuration yielded optical slice thickness of 0.8 μm allowing selective acquisition of the fluorescent signal from the nucleoplasm.

### Confocal Ca^2+^ imaging of cytoplasmic CaTs

Cells were loaded with the Ca^2+^-sensitive fluorescent indicator Fluo-4 (Molecular Probes, The Netherlands) as described previously [43] and imaged using an Olympus Fluoview 1000 confocal microscope (Olympus, USA) and the same excitation/emission settings as described in the section *Immunocytochemistry*. A 512-pixel scan line was positioned along the long axis of the myocyte and scanned every 1.27 ms. Consecutive scan lines were stacked over time and visualized as 2D image. For quantification of cytoplasmic CaTs cells were field-stimulated via two platinum electrodes at 0.2 or 1.5 Hz. A set of experiments was performed in the presence of Iso (1 µM).

### Analyses

Confocal images were analyzed by ImageJ software from NIH (http://rsbweb.nih.gov/ij/). The F_Nuc_/F_Cyto_ ratio was determined using freehand selection to draw the edge of the nucleus. Three measurements of the same size were taken from the surrounding cytosol, as well as one from the background (where no cells were located). The three cytosolic measurements were averaged, then the background fluorescence was subtracted from both the nuclear and cytosolic measurements, and finally the resulting nuclear fluorescence measure was divided by the cytosolic fluorescence measure (F_Nuc_/F_Cyto_). The total F_Nuc_/F_Cyto_ was determined for two groups of cells: those infected with HDAC4-GFP and those in which the endogenous localization of HDAC4 was examined using ICC. The mean was determined for both groups and as they showed identical ratio at baseline, they were pooled to determine the typical cellular distribution. Additionally, data in each graph was normalized to the control baseline F_Nuc_/F_Cyto_ ratio measured on a particular experimental day.

### Statistics

Statistics were performed with GraphPad Prism 8 (GraphPad Software, CA) using either the one-way Anova with the Newman–Keuls posttest, two-way ANOVA, or by the unpaired Student’s *t* test, where appropriate. Results are shown as the mean ± standard error of mean (SEM) with significance determined as two-tailed *p < 0.05, **p < 0.001 and ***p < 0.0001.

## Results

### Baseline HDAC4 nuclear localization in myocytes is predominantly regulated by CaMKII

**Fig. 1:**
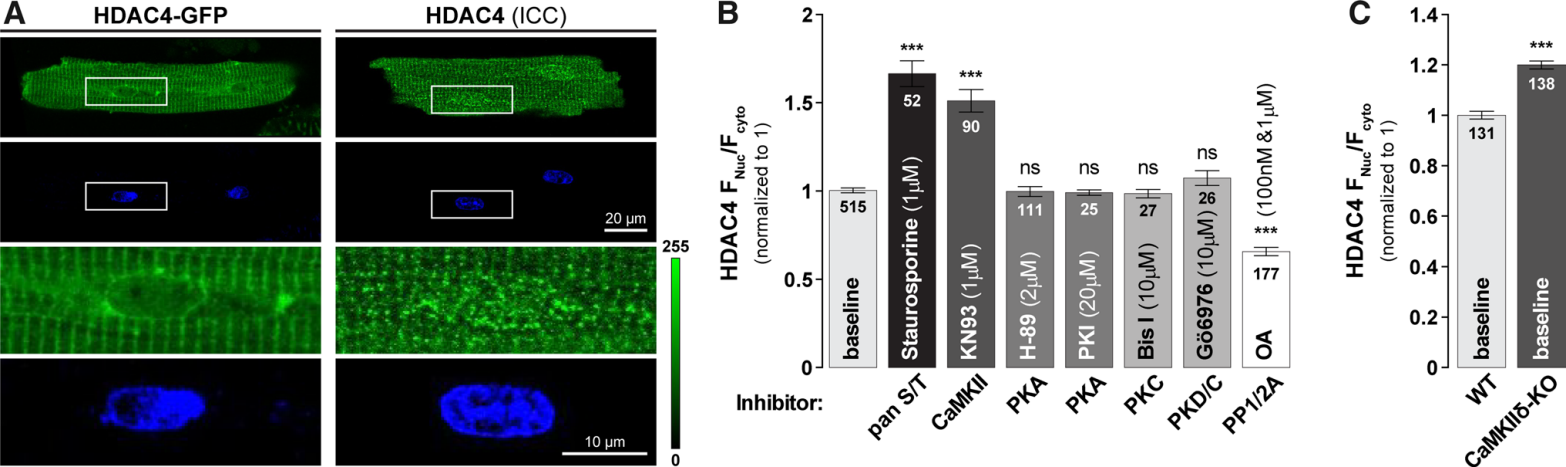
Baseline HDAC4 localization is governed by CaMKII. **a** Representative images of a live adult rabbit cardiomyocyte infected with HDAC4-GFP (*top left*) and the endogenous localization of HDAC4 in rabbit cardiomyocytes, detected by ICC (*top right*). Nuclear localization was confirmed by DAPI staining. Bottom panels represent zoom-in of corresponding cell nuclei. **b** Average values of HDAC4 nucleo-to-cytoplasmic fluorescence ratio F_Nuc_/F_Cyto_ in the absence or presence of specific inhibitors of cellular kinases or phosphatases. Cells were incubated with indicated drugs for 1 h at 37 °C. **c** Average values of HDAC4 F_Nuc_/F_Cyto_ ratio in WT and CAMKIIδ knockout (CAMKIIδ-KO) mice. ***p < 0.0001

Figure 1a shows representative confocal images of adult rabbit ventricular myocytes infected with HDAC4-GFP (*left*) and endogenous HDAC4 detected by immunocytochemistry (*right*) which were similar in cellular distribution. To determine the relative contribution of different kinases to baseline HDAC4 localization in cardiomyocytes at rest, we measured changes in the baseline ratio of nuclear-to-cytosolic HDAC4 (F_Nuc_/F_Cyto_) in the presence of selected kinase inhibitors (Fig. 1b). Cells treated with the pan-Ser/Thr kinase inhibitor staurosporine showed significantly higher nuclear HDAC4 (1.71 ± 0.04, *n* = 52 compared to baseline). Inhibition of CaMKII by KN93 also caused significant nuclear HDAC4 F_Nuc_/F_Cyto_ increase vs. baseline (1.42 ± 0.04, *n* = 90). Conversely, PKA inhibition by either commonly used, but less specific inhibitor H-89 or myristoylated highly specific peptide inhibitor PKI, failed to alter F_Nuc_/F_Cyto_ vs. baseline (1.02 ± 0.03, *n* = 111 and 0.98 ± 0.03, *n* = 25 of baseline, respectively). Thus basal PKA activity does not appear to influence HDAC4 localization in adult myocytes, likely due to low basal PKA activity [17, 57]. Because PKD [3, 13, 46] and PKC [30, 61] can mediate HDAC4 localization in neonatal myocytes, we also treated cells with either Gö6976 (a PKC and PKD inhibitor) or Bis I (a PKC inhibitor) for 1 h, without significant effects on baseline HDAC4 F_Nuc_/F_Cyto_ (Gö6976: 1.08 ± 0.03, *n* = 26 or Bis I: 0.97 ± 0.02, *n* = 27). Thus, neither PKC nor PKD directly alters baseline HDAC4 localization in quiescent adult myocytes. Finally, we treated cells with the phosphatase inhibitor, okadaic acid (OA) for 1 h to enhance the efficacy of any basal kinase activity (e.g., CaMKII) to cause HDAC4 phosphorylation. OA caused significant nuclear HDAC4 depletion (0.62 ± 0.04, *n* = 177), the expected effect of enhancing any basal CaMKII activity.

Although a potent inhibitor of CaMKII, KN93, is widely used as a research tool to test the functional role of CaMKII in cardiac physiology and pathology, it does have off-target effects. For example, KN93 was reported to inhibit voltage-gated potassium channels (including cardiac I_Kr_) [34, 56]. In addition, KN93 inhibits CaMKII activity by preventing CaM binding, but is poor at inhibiting autonomously active CaMKIIδ [22]. To most reliably confirm the critical role of CaMKII in regulating basal HDAC4 subcellular distribution, we further compared F_Nuc_/F_Cyto_ levels in WT vs. CaMKIIδ knockout (KO) mice that lack the predominant cardiac CaMKII isoform. As seen acutely with KN93, the CaMKIIδ-KO exhibited an elevated nucleo-to-cytoplasm HDAC4 ratio (1.20 ± 0.16, *n* = 138) vs. their WT littermates (*n* = 131, Fig. 1c).

Together, these results suggest that CaMKII is the primary modulator of HDAC4 localization in resting cardiomyocytes, despite the low basal activity of CaMKII expected in quiescent myocytes. That also explains that phosphatase inhibition enhanced the ability of modestly active CaMKII activity to drive HDAC4 nuclear export. The stronger nuclear accumulation induced by staurosporine may indicate that another unidentified kinases could also contribute to the baseline localization of HDAC4.

### CaMKII activation drives accumulation of HDAC4 in the cytoplasm

**Fig. 2:**
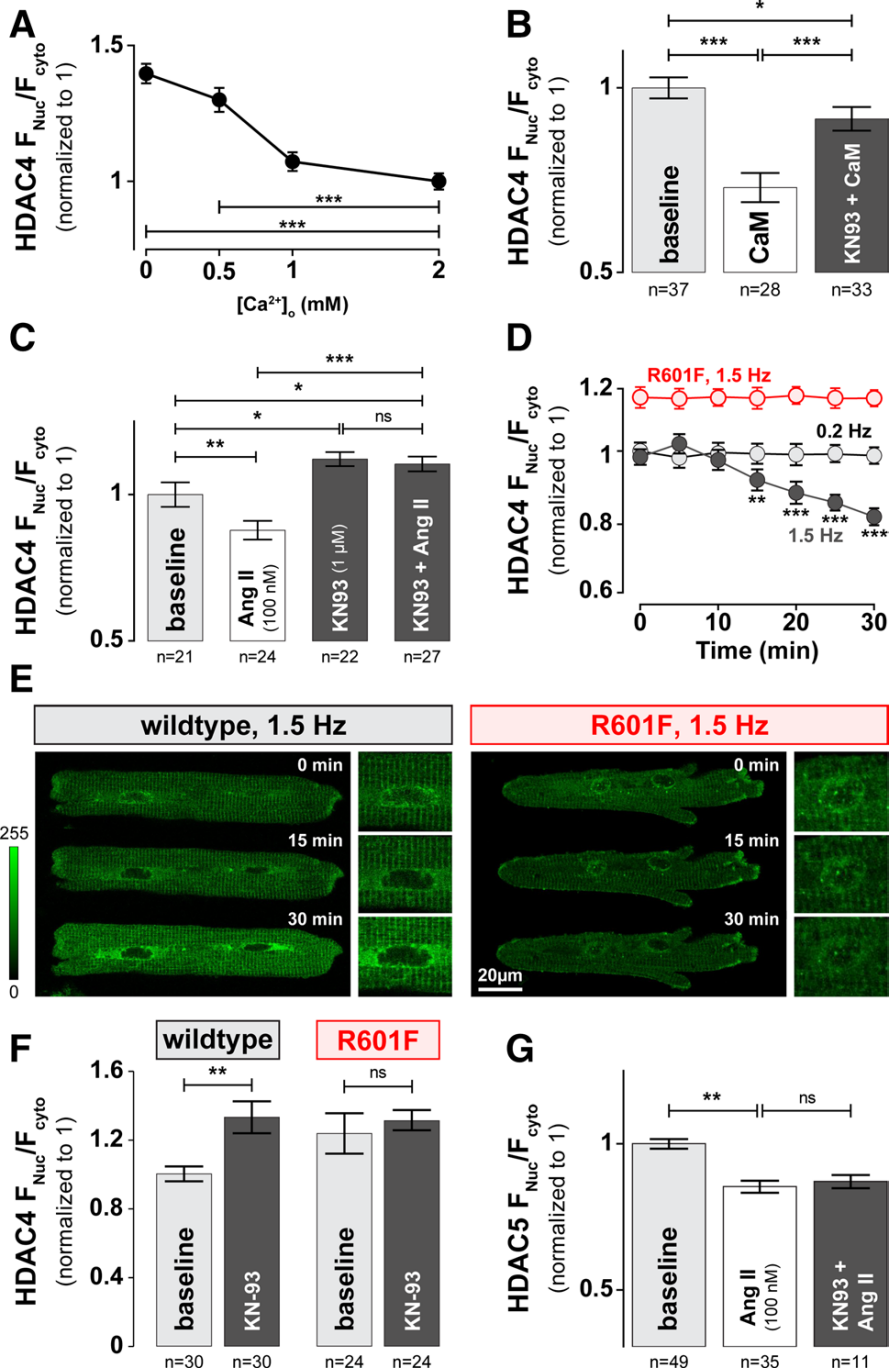
In rabbit ventricular myocytes, CaMKII activity promotes nuclear depletion of HDAC4. **a** Average values of HDAC4 nucleo-to-cytoplasmic fluorescence ratio in quiescent cells incubated in Normal Tyrode solution containing 0, 0.5, 1 and 2 mM Ca^2+^. **b** Average values of HDAC4 nucleo-to-cytoplasmic fluorescence ratio (F_Nuc_/F_Cyto_) in cells overexpressing calmodulin (CaM) with and without preincubation with KN93. **c** Average values of HDAC4 nucleo-to-cytoplasmic fluorescence ratio in cells treated with 100 nM AngII for 30 min in the presence or absence of KN93**. d** Time course of wild type (*grey*) or R601F (*red*) HDAC4-GFP translocation measured as F_Nuc_/F_Cyto_ in response to low (*light*) or high (*dark*) pacing frequency (*n* = 20–24 cells per group). **e** Representative confocal images of cultured adult cardiomyocyte infected with wild type or R601F HDAC4-GFP, paced at 1.5 Hz for 0, 15 or 30 min. **f** Average values of HDAC4 F_Nuc_/F_Cyto_ ratio in cells overexpressing wild type or R601F HDAC4-GFP with or without preincubation with KN93. **g** Average values of HDAC5 F_Nuc_/F_Cyto_ in cells treated with 100 nM AngII for 30 min in the absence or presence of KN93. ***p < 0.0001, **p < 0.001, *p < 0.01

We next assessed the effects on HDAC4 localization of stimulation strategies that are known to further promote CaMKII activation (Fig. 2). Since increasing [Ca^2+^] can increase CaMKII activity [44, 45] we incubated quiescent adult rabbit cardiomyocytes for 1 h at 37 °C with Normal Tyrode’s solution (NT) containing different [Ca^2+^] (Fig. 2A). Higher extracellular Ca^2+^ concentration ([Ca^2+^]_o_) led to significant HDAC4 nuclear depletion (e.g., 1.00 ± 0.03 at 2 mM Ca^2+^ and 1.40 ± 0.04 at 0 mM Ca^2+^; *p* < 0.0001). This [Ca^2+^]-dependent shift is consistent with our data in Fig. 1, with respect to CaMKII-dependent modulation of HDAC4, even in quiescent myocytes.

Availability of Ca^2+^/CaM may limit activity of CaMKII [45] and CaM levels decline during the normal culture process (Supp. Figure 1) so we next overexpressed CaM, which resulted in enhanced nuclear export of HDAC4 at rest (F_Nuc_/F_Cyto_ = 0.73 ± 0.03, *n* = 28; Fig. 2b). Pretreating cells with KN93 to inhibit CaMKII, significantly reduced the CaM-induced shift in HDAC4 localization (0.92 ± 0.03, *n* = 33) indicating the effect is mainly CaMKII- as opposed to CaM-dependent.

Angiotensin II (Ang II) is G-protein coupled receptor pathway known to activate CaMKII in adult ventricular myocytes [21, 22]. Treatment with 10 μM Ang II resulted in significant nuclear depletion of HDAC4 (F_Nuc_/F_Cyto_ = 0.81 ± 0.04, *n* = 56), while pretreating cells with KN93 prior to Ang II exposure, prevented the Ang II-induced effect (1.10 ± 0.03, *n* = 27; Fig. 2c).

We previously showed that simply increasing myocyte pacing frequency activates CaMKII and nuclear export of HDAC4 [42]. In agreement with this, we observed a time-dependent reduction in the wildtype HDAC4-GFP F_Nuc_/F_Cyto_ ratio when pacing was switched from 0.2 to 1.5 Hz (Fig. 2d). In contrast, when cells were infected with mutant R601F-HDAC4-GFP that is unable to bind CaMKII, increased pacing frequency failed to drive HDAC4 out of the nucleus (Fig. 2d, e). Also, while KN93 significantly increased F_Nuc_/F_Cyto_ levels in cells expressing wildtype HDAC4, it did not alter distribution of R601F-HDAC4 (Fig. 2f). Note also that the baseline F_Nuc_/F_Cyto_ levels of R601F-HDAC4 were similar to those of the wildtype HDAC4 treated with KN93.

HDAC5 is a related member of class IIa HDACs and shares significant sequence homology with HDAC4, but it notably lacks the CaMKII-docking site of HDAC4 [4]. Cells infected with HDAC5-GFP showed a similar response to treatment with Ang II (0.85 ± 0.02, *n* = 35; Fig. 2g). However, that might, in principle be, due caused by oligomerization with HDAC4 [2]. However, when HDAC5-infected cells were pretreated with 1 μM KN93 and then exposed to Ang II, the enhanced nuclear export was not reversed (0.87 ± 0.02, *n* = 19). These observations contrast those with HDAC4, suggesting a more prominent role for CaMKII in HDAC4 vs. HDAC5 nuclear export (for which PKD may be a more prominent activator).

### β-AR activation drives nuclear HDAC4 accumulation and requires S265/266

**Fig. 3:**
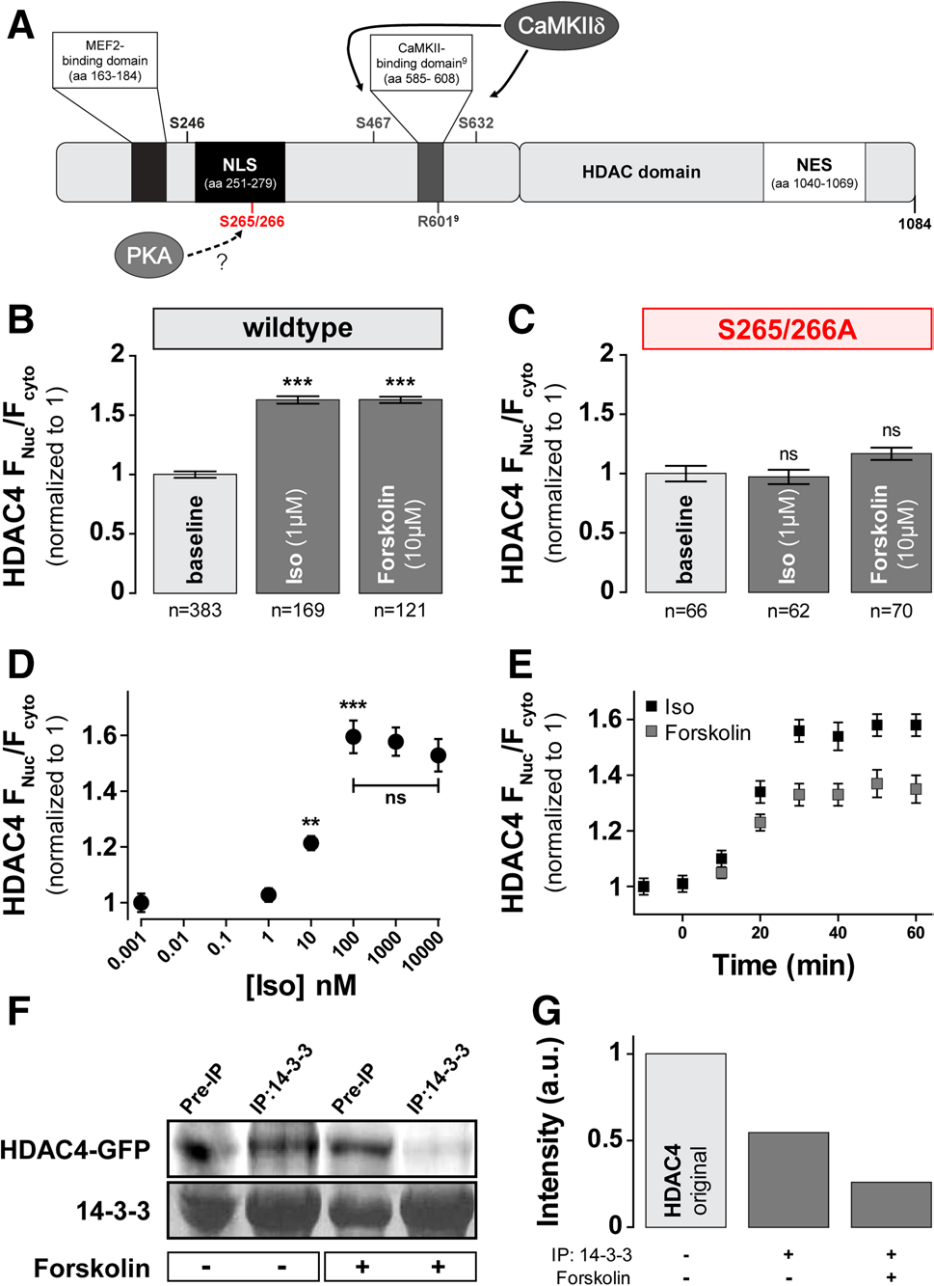
β-AR activation in rabbit myocytes induces nuclear accumulation of HDAC4 and requires S265/266. **a** HDAC4 sequence with selected domain and residues indicated. NLS = nuclear localization sequence, NES = nuclear export signal. **b** Average values of HDAC4 nucleo-to-cytoplasmic fluorescence ratio of endogenous HDAC4 or cells infected with HDAC4-GFP (polled data) and treated for 30–60 min with either 1 μM Iso or 10 μM forskolin at 37 °C. **c** Average values of HDAC4 nucleo-to-cytoplasmic fluorescence ratio of cells infected with HDAC4-S265/266A-GFP and treated as in (**b**). **d** Dose-dependent accumulation of HDAC4 in the nucleus upon Iso treatment. Each point represents an *n* = 22–26. **e** Time course of HDAC4 redistribution in response to Iso (*black*) or forskolin (*grey*). Each point represents an *n* = 20–25. Representative example (**f**) and quantification (**g**) of immunoprecipitation with a 14–3-3 antibody and a GFP antibody to detect the HDAC4–14–3-3 complex with or without forskolin treatment. ***p < 0.0001

To understand how HDAC4 is modulated by sympathetic stimulation via β-adrenergic receptors (β-AR), we treated cells with 1 μM Iso (Fig. 3b). We also treated cells with the adenylate cyclase (AC) activator, forskolin (10 μM), to directly activate cAMP-production independent of β-AR [25]. Treatment with either Iso or forskolin causes substantial and identical nuclear accumulation of HDAC4 (1.62 ± 0.03, *n* = 169 or 121, respectively) (Fig. 3b). Notably, this PKA-induced nuclear import is opposite to that observed upon CaMKII activation, and so it seems likely that alternative phosphorylation sites are involved.

Based on a regulatory phosphorylation site identified in the NLS domain of HDAC5 (S279/S280) that influences HDAC5 nuclear import [12, 29], our predictions using NetPhos 2.0 and NetPhosK 1.0 [7, 8], we hypothesized that HDAC4 S265/266 might be crucial for PKA-dependent HDAC4 nuclear import [36, 40]. (De)-phosphorylation at these sites within the NLS (Fig. 3a) might influence the interaction of phospho-HDAC4 with chaperone 14-3-3, possibly masking a NES [29, 62]. To test if intact S265/266 motif is required for nuclear accumulation upon Iso or forskolin treatment, we repeated these experiments with a mutant HDAC4 where both S265 and S266 were mutated to alanine (HDAC4-S265/266A-GFP) (Fig. 3c). At baseline HDAC4-S265/266A-GFP distribution was not different from WT HDAC4 (*not shown*), consistent with a lack of baseline PKA effect as shown in Fig. 1b. However, the S265/266A HDAC4 mutant showed no significant response to either Iso or even forskolin treatment (0.97 ± 0.06, *n* = 62 or 1.17 ± 0.05, *n* = 70, respectively). Thus, the S265/266 sites are required for PKA-mediated nuclear accumulation of HDAC4 under β-AR stimulation and increased cAMP levels in adult ventricular myocytes.

We further studied the concentration dependence of the Iso effect by treating myocytes with increasing Iso concentrations for 1 h (Fig. 3d). Net HDAC4 nuclear translocation was apparent at 10 nM Iso and reached maximum at 100 nM. The time course of nuclear accumulation of HDAC4 in cells treated with 1 μM Iso or 10 µM forskolin revealed significant nuclear accumulation at 20 min time point, reaching a plateau at 30–60 min (Fig. 3e).

Finally, to test our hypotheses that increased cAMP level leads to nuclear entrapment by preventing HDAC4 association with 14-3-3, we assessed 14–3-3 co-immunoprecipitation with HDAC4-GFP in myocytes exposed to 10 μM forskolin for 1 h and compared it to untreated controls. 14-3-3 immunoprecipitates were probed with GFP (for HDAC4 presence) and 14–3-3 antibodies. Compared to the total HDAC4 in the original sample, only a portion of the available HDAC4 (possibly the S246/467/632 phosphorylated fraction) was immunoprecipitated with 14–3-3 (Fig. 3f, g). Treatment with forskolin led to a further reduction in the amount of HDAC4 associated with 14-3-3. Therefore, forskolin treatment decreases the interaction between HDAC4 and 14-3-3, resulting in the nuclear retention of HDAC4.

### PKA is responsible for HDAC4 nuclear accumulation in response to β-AR stimulation

**Fig. 4:**
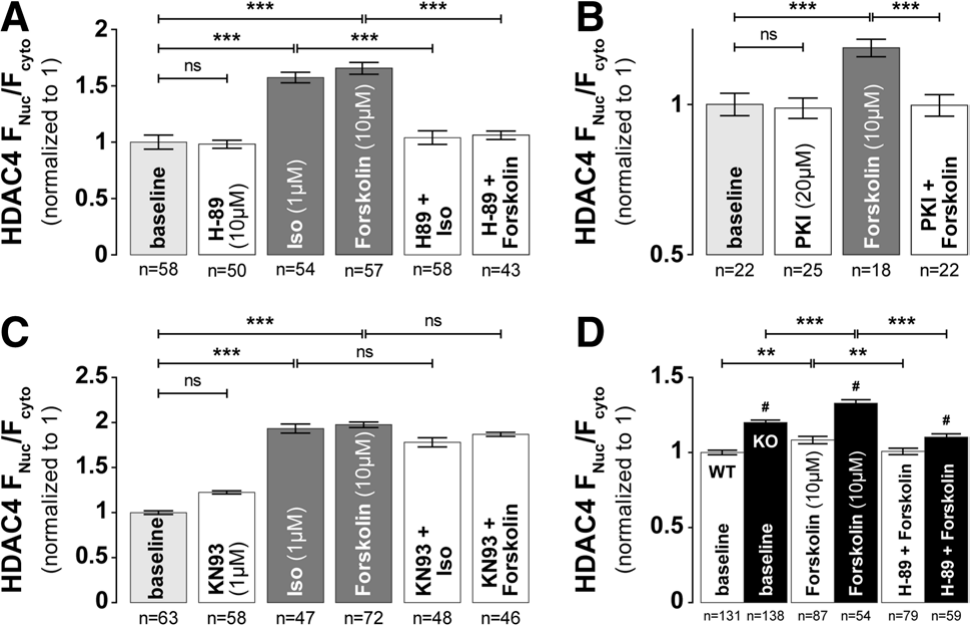
Iso- or forskolin-induced HDAC4 nuclear accumulation is PKA-dependent. Average values of HDAC4 F_Nuc_/F_Cyto_ under PKA signaling agonists in the absence or presence of specific PKA inhibitor H-89 (**a**) or PKI (**b**). Cells were incubated with indicated drugs for 30 min at 37 °C. **c** Average values of HDAC4 F_Nuc_/F_Cyto_ ratio under PKA signaling agonists with or without 30 min preincubation with specific CaMKII inhibitor KN93. **d** Average values of HDAC4 F_Nuc_/F_Cyto_ under PKA signaling agonists in the absence or presence of H-89 in WT and CAMKIIδ knockout (KO) mice. ***p < 0.0001 and **p < 0.001 vs. baseline; ^#^*p* < 0.01 vs. WT

Next, we tested whether PKA was the kinase responsible for the Iso and forskolin-induced HDAC4 import via interaction with S265/266. We pre-treated myocytes with 10 μM H-89, a concentration that can inhibit PKA activity under stimulation of either Iso or forskolin [70]. Similar to the results with 2 μM H-89 (Fig. 1b), 10 μM H-89 did not affect the baseline distribution of HDAC4 (0.98 ± 0.04, *n* = 50), but fully prevented Iso- and forskolin-induced HDAC4 nuclear accumulation (1.04 ± 0.06, *n* = 58, and 1.06 ± 0.04, *n* = 43, respectively) (Fig. 4a), indicating that the HDAC4 nuclear import induced by β-AR stimulation is mainly PKA-dependent. To confirm adequate specificity of PKA inhibition, we also treated cells with 20 μM myristoylated PKI prior to stimulation with forskolin (Fig. 4b) and—as expected—forskolin-induced effect was abolished (1.00 ± 0.04, *n* = 22).

Since β-AR stimulation can also activate CaMKII [21, 25], independent of PKA [53], we repeated the experiments after pretreatment with KN93. Even in the presence of KN93, both Iso and forskolin robustly increased the F_Nuc_/F_Cyto_ ratio, (1.93 ± 0.03, *n* = 47 and 1.78 ± 0.02, *n* = 72, respectively) and not significantly different from Iso or forskolin alone (Fig. 4c). We also tested the forskolin-induced response in CaMKIIδ-KO mice. Although baseline F_Nuc_/F_Cyto_ ratio was higher in CaMKIIδ-KO mice, forskolin still caused a further increase that was sensitive to PKA inhibition by H-89 (Fig. 4d). Together, these data indicate that PKA is predominately responsible for the effects seen under Iso and forskolin stimulation. Although CaMKII can be activated by Iso or cAMP (via Epac [53]) and would drive HDAC4 in the opposite direction as PKA, these results suggest that the PKA-mediated effects on HDAC4 may dominate over those of CaMKII under predominant β-AR stimulation.

### PKA-dependent nuclear accumulation of HDAC4 dominates CaMKII-dependent export

**Fig. 5:**
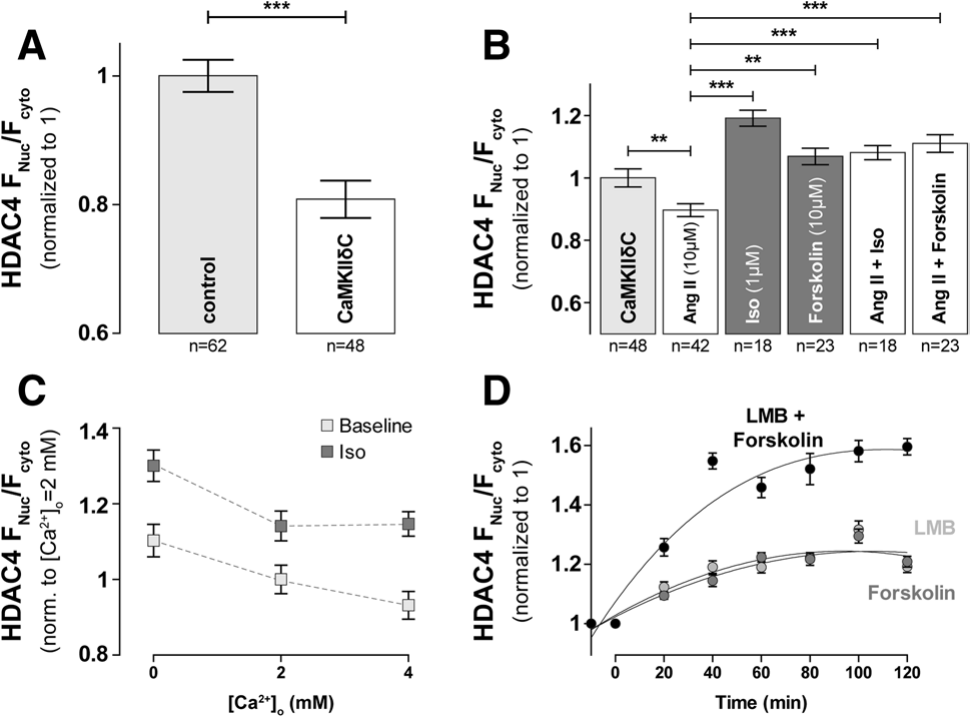
PKA-dependent nuclear accumulation of HDAC4 is dominant over CaMKII-dependent cytosolic accumulation. **a** Average values of HDAC4 F_Nuc_/F_Cyto_ in control rabbit myocytes and myocytes after overexpression of CaMKIIδC. **b** Average values of HDAC4 F_Nuc_/F_Cyto_ in the absence or presence of specific agonists of PKA and CaMKII signaling. Myocytes were incubated with indicated drugs for 30–60 min at 37 °C. **c** Average values of HDAC4 F_Nuc_/F_Cyto_ in quiescent cells incubated for 1 h in Normal Tyrode solution containing 0, 2 and 4 mM [Ca^2+^]° in the presence or absence of Iso. **d** Time course of HDAC4 redistribution in response to forskolin (*dark grey*), LMB (*light grey*) or both (*black*). Images were taken every 20 min to assess maximal nuclear accumulation as well as the time to reach half-max. Each point represents an *n* = 22–27. ***p < 0.0001, **p < 0.001

To test how the relative dominance of the opposing effects of agonist-induced PKA activity (nuclear retention) or CaMKII activity (nuclear depletion), we first overexpressed CaMKIIδC in cardiomyocytes and treated them with PKA and CaMKII agonists. Under the same experimental conditions we previously found exogenous CaMKIIδ to be 63.2 ± 33% of endogenous CaMKII [68], which is comparable to the increase seen in heart failure [41]. The overexpression of CaMKIIδC (as occurs in HF) by itself led to significant nuclear export of HDAC4 (F_Nuc_/F_Cyto_ decreased from 1 ± 0.02, *n* = 84 to 0.83 ± 0.02, *n* = 70; Fig. 5a), consistent with a substantial increase in the basal CaMKII-dependent nuclear export. Then, in Fig. 5b all cells overexpressed CaMKIIδC, so the data was now normalized to untreated cells overexpressing CaMKIIδC (1 ± 0.02, *n* = 70). Stimulation with 10 μM Ang II caused further nuclear depletion of HDAC4 (0.83 ± 0.02, *n* = 62), while treatment with Iso still drove nuclear import (Fig. 5b). When cells were first treated with 10 μM Ang II and then exposed to Iso, a 23% increase in F_Nuc_/F_Cyto_ ratio compared to Ang II alone was observed. Importantly, this increase is similar to the 19% increase induced by Iso alone in the CaMKII overexpressing myocytes. Similar results were obtained with forskolin alone or when cells were pretreated with Ang II (Fig. 5b). These results indicate that regardless of whether CaMKII has been already activated, when PKA is also activated, the PKA-mediated effects dictate more potently the localization of HDAC4. To determine if PKA-mediated nuclear retention of HDAC4 was modified by changes in [Ca^2+^]_o_ which could alter CaMKII activity, we incubated cardiomyocytes in NT solution containing different [Ca^2+^]_o_ for 1 h in the presence or absence of 1 µM Iso (Fig. 5c). Treatment with Iso led to significant nuclear accumulation of HDAC4 (*p* < 0.0001) and the Ca^2+^-dependent reduction in F_Nuc_/F_Cyto_ was blunted at higher [Ca^2+^]_o_, consistent again with some relative dominance of the PKA vs. CaMKII effect on HDAC4 nuclear levels.

### β-AR stimulation drives nuclear accumulation and blocks nuclear export of HDAC4

The reduced 14-3-3 co-immunoprecipitation experiments (Fig. 3f, g) suggest that PKA inhibits HDAC4 export, but it could also influence HDAC4 nuclear import. To investigate whether PKA alters HDAC4 nuclear import we used leptomycin B (LMB) to block nuclear export (Fig. 5d) to allow monitoring of unidirectional nuclear import. LMB modifies CRM-1 [48], preventing its nuclear export by inhibiting the association between CRM-1 and the NES of the CRM-1-dependent cargo (HDAC4 in this case). Blocking nuclear export with 10 nM LMB caused nuclear HDAC4 accumulation, reaching half-maximum at 18 min and plateau at F_Nuc_/F_Cyto_ at 40–50 min (Fig. 5d). This rise largely reflects the baseline level of HDAC4 nuclear import (although not exclusively unidirectional at early time points). Forskolin alone produces a very similar HDAC4 nuclear accumulation time course, which would be consistent with PKA-dependent HDAC4 phosphorylation strongly inhibiting nuclear export. However, when forskolin and LMB were added together, the rate and extent of nuclear HDAC4 accumulation was greatly enhanced vs. either treatment alone. This observation suggests that forskolin not only slows nuclear export of HDAC4 but also enhances nuclear import of HDAC4, which may be a consequence of altered phosphorylation of S265/266 within the NLS (Fig. 3a).

### PKA and CaMKII have different temporal impacts on HDAC4 translocation

**Fig. 6:**
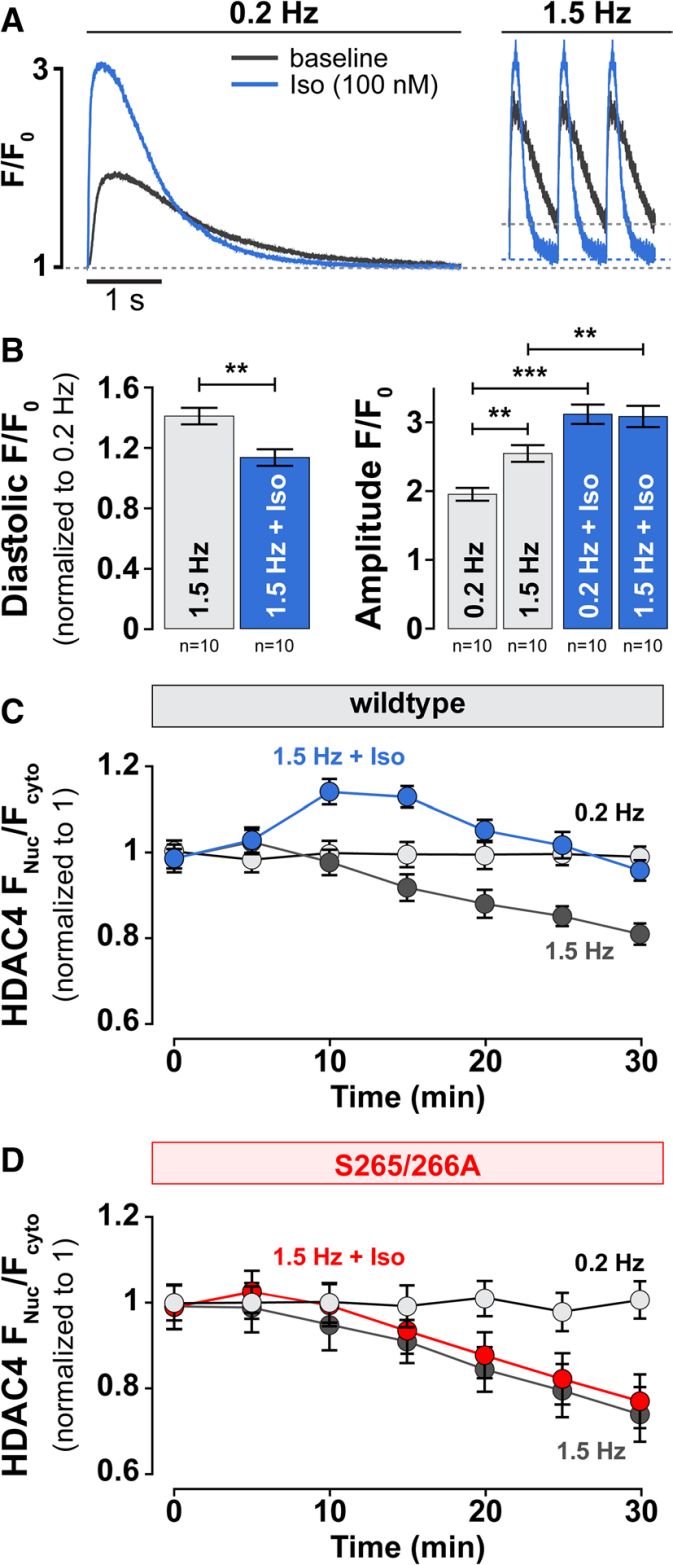
PKA and CaMKII have different time course of action on HDAC4 redistribution. **a** Original recordings of cytoplasmic [Ca^2+^] transients in isolated ventricular rabbit cardiomyocytes during increases of stimulation frequency from 0.2 to 1.5 Hz at baseline (*grey*) and in the presence of 100 nM Iso (*blue*). **b** Corresponding average values of diastolic [Ca^2+^] (*left*) and amplitude (*right*) of cytoplasmic [Ca^2+^] transients. **c** Time course of HDAC4-GFP translocation measured as F_Nuc_/F_Cyto_ in response to pacing frequency at baseline (*grey*) and in the presence of 100 nM Iso (*blue*). **b**, **c** Each point represents an *n* = 20–24. **d** Time course of HDAC4-GFP translocation measured as F_Nuc_/F_Cyto_ in response to pacing frequency at baseline (*grey*) and in the presence of 100 nM Iso (*red*). Each point represents an *n* = 8–10. ***p < 0.0001, **p < 0.001

The normal Ca^2+^ transient cycle governs myocyte contraction and relaxation. This may influence dynamic activation of cellular kinases (especially CaMKII), as well as the relative contribution of CaMKII (and PKA) to the overall HDAC4 redistribution vs. quiescent myocytes. Increasing pacing rate increases the Ca^2+^ transient amplitude, frequency, diastolic and time-averaged [Ca^2+^]_i_ (Fig. 6a, b), all of which increase CaMKII activation and HDAC4 nuclear export [42]. At very low pacing frequency (0.2 Hz) there was negligible HDAC4 nuclear export, but increasing pacing to 1.5 Hz caused progressive net HDAC4 nuclear export, consistent with a CaMKII-dependent shift (Fig. 6c) that required CaMKIIδ docking to HDAC4 (Fig. 2d). As expected, application of Iso (100 nM) resulted in a robust increase of cytoplasmic [Ca^2+^] transients at both frequencies studied (Fig. 6a, b). However, when 1.5-Hz pacing was initiated with Iso application, there was transient, nuclear HDAC4 accumulation (during the first 15 min), but that reversed during the next 15 min, in parallel to the strict pacing-induced CaMKII-dependent HDAC4 export (Fig. 6c). This differs from the prolonged stable rise in F_Nuc_/F_Cyto_ seen with Iso or forskolin in quiescent myocytes (Fig. 3e). Thus, the PKA-dependent HDAC4 nuclear import dominates at short times, but appears to be overridden by CaMKII-dependent nuclear export at longer times. Importantly, in a subset of experiments with S265/266A mutant, an immediate decrease in F_Nuc_/F_Cyto_ was observed upon increased stimulation frequency, with slightly steeper decline rate, suggesting the essential role of the S265/266 motif in the observed acute PKA effect on HDAC4 nuclear import (Fig. 6d).

### The co-regulation of CaMKII and PKA signaling is altered in experimental heart failure

**Fig. 7:**
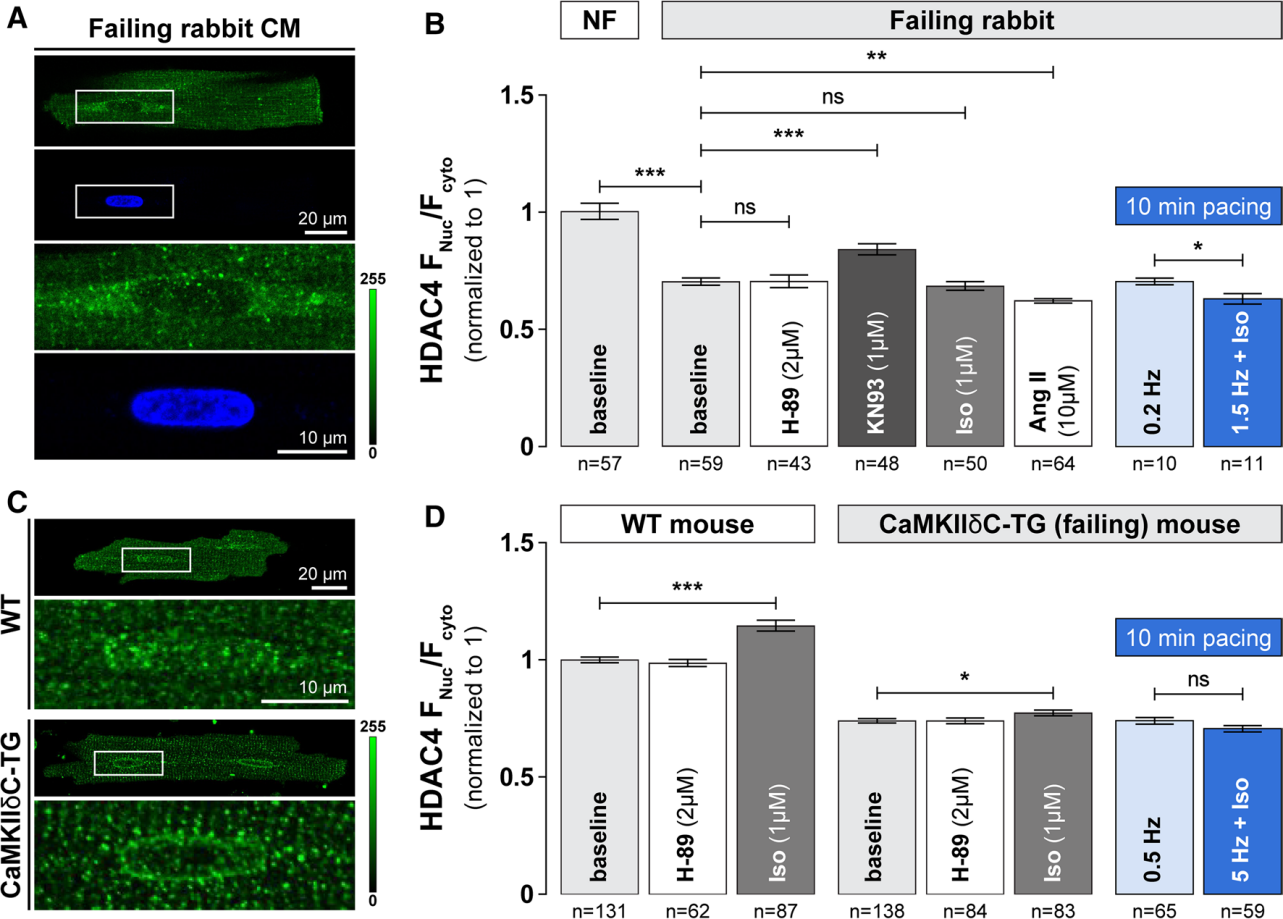
Co-regulation of HDAC4 localization by PKA and CaMKII is altered in heart failure. **a** Representative images of the endogenous localization of HDAC4 in failing rabbit cardiomyocytes, detected by ICC (*top*). Nuclear localization was confirmed by DAPI staining. Bottom panels represent zoom-in of corresponding cell nuclei. **b** Average values of HDAC4 nucleo-to-cytoplasmic fluorescence ratio (F_Nuc_/F_Cyto_) in the absence or presence of specific inhibitors/activators of cellular kinases without (*gray*) or with (*blue*) 10 min electrical stimulation of 0.2 or 1.5 Hz. NF—non-failing. Cells were incubated with indicated drugs for 1 h at 37 °C. **c** Representative images of the endogenous localization of HDAC4 in cardiomyocytes isolated from wild type (WT; *top*) or CaMKIIδC transgenic (CaMKIIδC-TG; *bottom*) mice, detected by ICC. **d** Average values of HDAC4 F_Nuc_/F_Cyto_ ratio in WT and CAMKIIδC-TG mice in the absence or presence of specific inhibitors/activators of protein kinase A without (*gray*) or with (*blue*) 10 min electrical stimulation of 0.2 or 1.5 Hz. Cells were incubated with indicated drugs for 1 h at 37 °C. ***p < 0.0001. ***p < 0.001, *p < 0.05

The disbalance in PKA vs. CaMKII-mediated signaling is well documented in experimental and human heart failure, with increased baseline CaMKII activity [23, 41, 59] and diminished expression of β_1_-AR [10, 18]. We next tested whether this PKA vs. CaMKIIδ disbalance at HDAC4 is altered in two animal models of heart failure; a non-ischemic rabbit heart failure model and CaMKIIδC transgenic mice (Fig. 7). Baseline HDAC4 F_Nuc_/F_Cyto_ was dramatically reduced in resting myocytes isolated from either failing rabbit hearts (Fig. 7a, b) or 10–12 weeks old CaMKIIδC transgenic mice (Fig. 7c, d), for which we previously documented overt heart failure [41]. While the regulation of HDAC4 localization at baseline was primarily regulated by CaMKII as was observed in non-failing hearts, nuclear accumulation of HDAC4 upon 1 µM Iso treatment was strikingly inhibited/attenuated in failing quiescent and electrically stimulated cardiomyocytes. One somewhat unexpected finding was that KN93 was less able to increase nuclear HDAC4 accumulation in failing vs. non-failing cells, because baseline CaMKII activation is increased in both heart failure models [41, 42]. This may be because KN93 is less able to inhibit autonomously activated CaMKII (which occurs in HF), but might also reflect other long-term remodeling processes that occur during heart failure development and progression.

### HDAC4 localization in human hearts follows similar regulation pattern

**Fig. 8:**
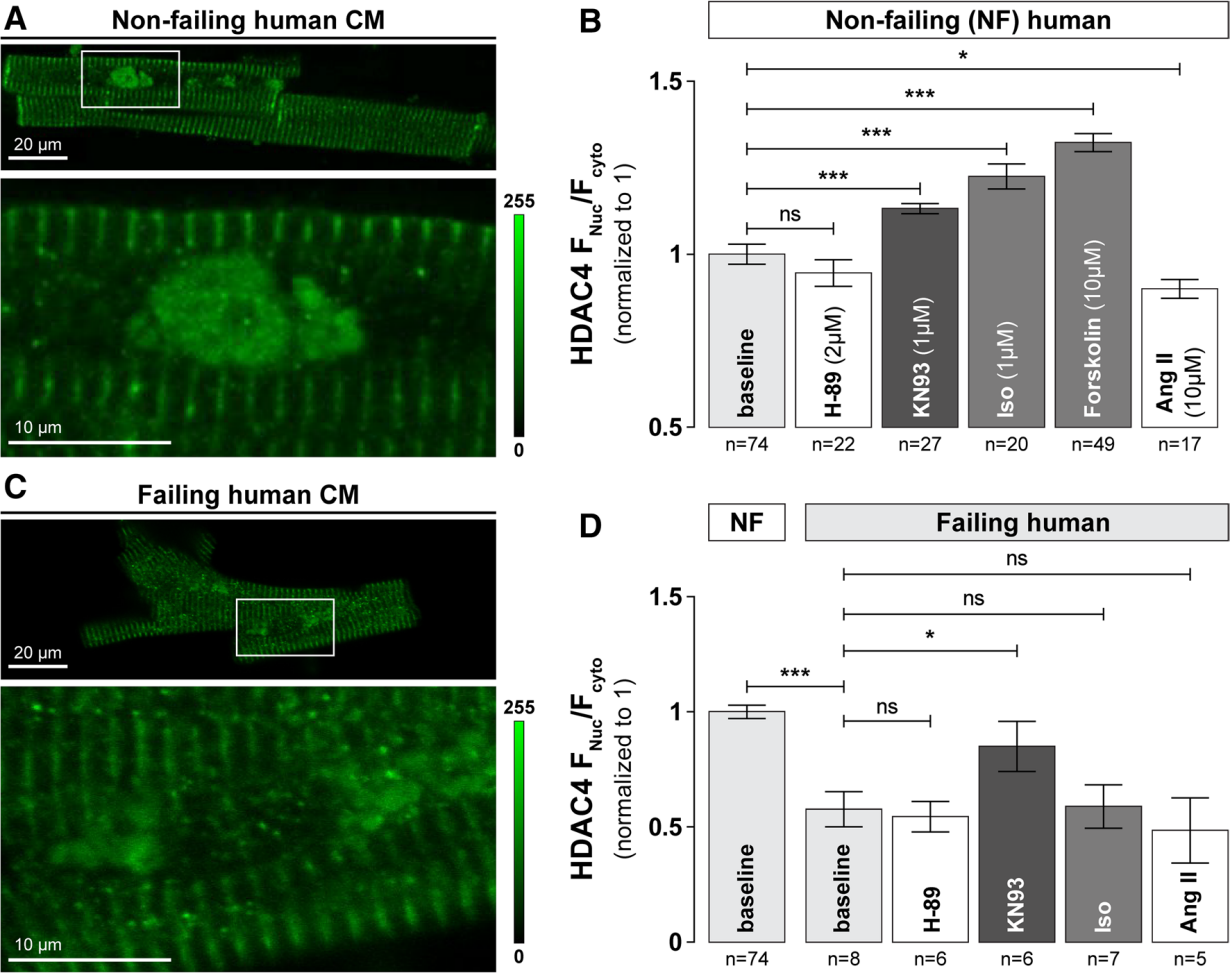
HDAC4 localization in human ventricular myocytes is regulated in similar manner. **a** Representative image of endogenous HDAC4 localization in cardiomyocytes isolated from non-failing human heart, detected by ICC. Bottom panel represent zoom-in of corresponding cell nuclei. **b** Average values of HDAC4 F_Nuc_/F_Cyto_ ratio in non-failing (NF) human myocytes in the absence or presence of specific agonists or antagonists of PKA or CaMKII signaling. **c** Representative image of endogenous HDAC4 localization in cardiomyocytes isolated from failing human heart, detected by ICC. Bottom panel represent zoom-in of corresponding cell nuclei. **d** Average values of HDAC4 F_Nuc_/F_Cyto_ ratio in failing human myocytes in the absence or presence of specific agonists or antagonists of PKA or CaMKII signaling. Cells were incubated with indicated drugs for 1 h at 37 °C. ***p < 0.0001, *p < 0.01

We were able to obtain non-failing and failing adult human heart tissue from which we isolated ventricular myocytes to test whether HDAC4 localization (determined by ICC) was similar to what we found in rabbit ventricular myocytes (Fig. 8a, b). Human myocytes had somewhat higher mean baseline F_Nuc_/F_Cyto_ (~ 18%) compared to rabbit myocytes. However, HDAC4 in quiescent human myocytes responded similarly to rabbits, showing no significant response when exposed to H-89 (0.95 ± 0.04, *n* = 22) but significant nuclear accumulation upon treatment with KN93 (1.13 ± 0.01, *n* = 27). Likewise, the Iso- and forskolin-induced increase in nuclear HDAC4 in human cardiomyocytes mirrored what we observed in rabbits (F_Nuc_/F_Cyto_ of 1.22 ± 0.04, *n* = 20 and 1.32 ± 0.03, *n* = 49, respectively). CaMKII activation via Ang II also led to nuclear depletion of HDAC4, as in rabbits (0.89 ± 0.03, *n* = 17). Importantly, the shifts in CaMKII and PKA regulation of F_Nuc_/F_Cyto_ in the rabbit heart failure model (Fig. 7a), were recapitulated in myocytes isolated from failing human hearts (e.g., lower baseline, loss of Iso effect, weaker Ang II effect; Fig. 8c, d). We conclude that regulation of nucleocytoplasmic shuttling of HDAC4 in response to PKA and CaMKII signaling in adult human ventricular myocytes and the shift towards predominant CaMKII regulation in failing cells is qualitatively similar to the shuttling mediated in rabbits, underscoring the clinical importance of the present work.

## Discussion

**Fig. 9:**
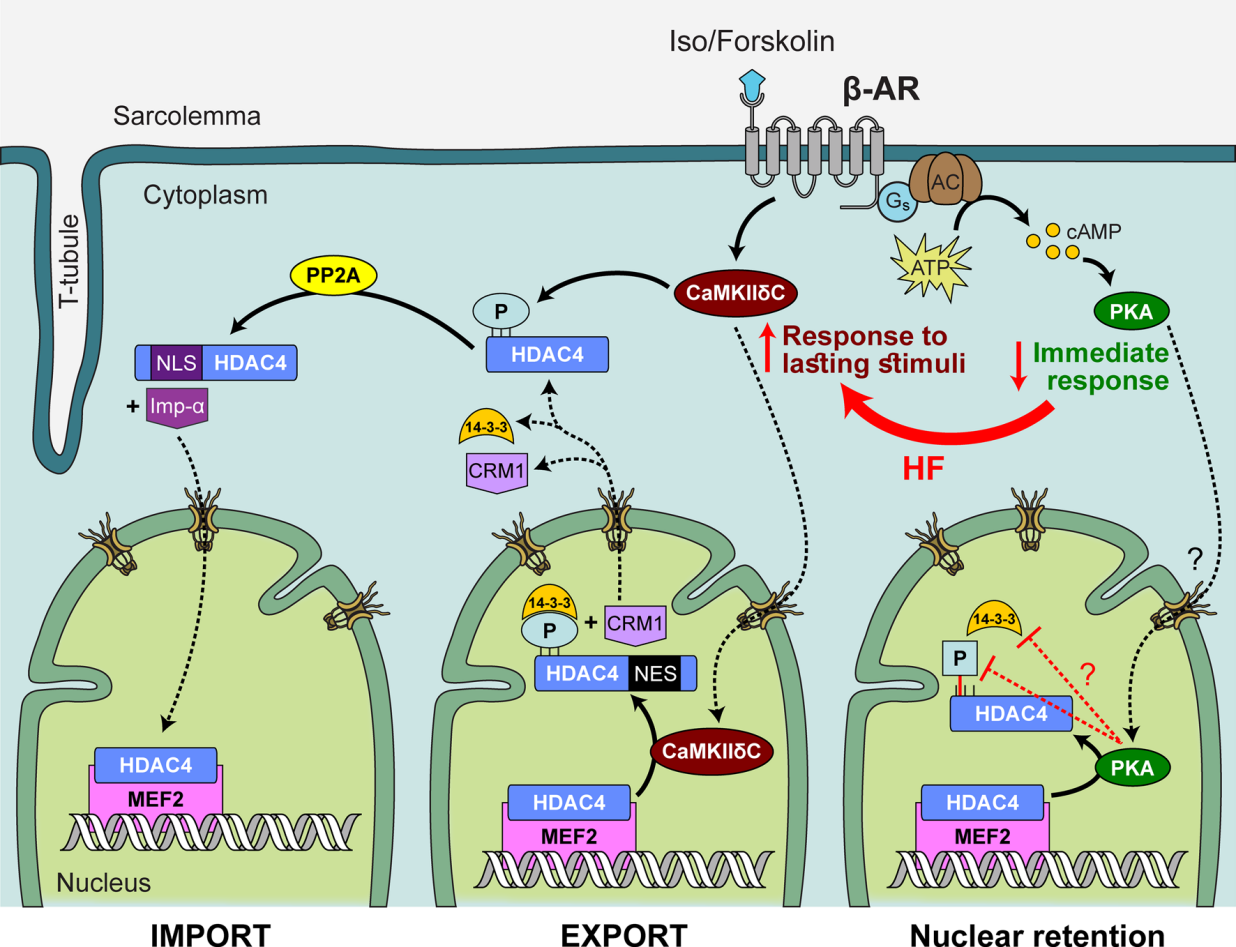
HDAC4 nucleo-cytoplasmic shuttling is differentially controlled by PKA and CaMKII. *Left Panel*: Cytosolic, dephosphorylated HDAC4 has an NLS recognized by importin-α, leading to nuclear import of HDAC4 via the nuclear pore complex. Once in the nucleus, HDAC4 can complex with MEF2 allowing for both the deacetylation of histone tails within nucleosomes, as well as preventing HATs (like p300) from complexing with MEF2. The HDAC4-MEF2 complex plays a role in preventing reactivation of the fetal gene program by maintaining the relevant DNA in a condensed (and therefore inaccessible) chromatin conformation. *Middle Panel*: HDAC4 can be phosphorylated by kinases including CaMKII and PKA. CaMKII contributes to the phosphorylation of Ser 467 and 632, whereupon chaperone protein 14-3-3 recognizes the phosphoserines and binds to them. This 14-3-3 binding masks the NLS and helps expose the NES, which can bind the exportin CRM-1, leading to nuclear export. Once in the cytosol, 14-3-3 and CRM-1 can dissociate leaving phosphorylated HDAC4 which is not available to be imported unless dephosphorylated by phosphatases like PP2A. Additionally, cytosolic, dephosphorylated HDAC4 can also be phosphorylated by cytosolic CaMKII which thereby prevents nuclear import. HDAC4 located in the cytoplasm leaves MEF2 available to bind HATs, which can lead to a more open chromatin conformation and activation of the fetal gene program. *Right Panel*: Nuclear localization of HDAC4 can also be influenced by PKA via its effect on the critical residues S265/266. These serines are located within the NLS and alteration in their phosphorylation may prevent the binding of 14–3-3 and therefore the nuclear export of HDAC4. Activation of PKA via β-AR stimulation is necessary for S265/266 dephosphorylation and leads to the nuclear accumulation of HDAC4, allowing for a close chromatin conformation. *Red Arrows:* In heart failure (HF), the orchestrated co-regulation of HDAC4 localization by PKA and CaMKII is shifted, in such way that CaMKII-dependent effects dominate PKA-dependent response, overall favoring nuclear export of HDAC4

Establishing the mechanisms that determine HDAC4 localization in adult cardiomyocytes has significant translational implications due to the role of class II HDACs in the development of heart failure [14]. In this study, we directly measured HDAC4 translocation in adult ventricular myocytes from mouse, rabbit and human hearts and we demonstrated for the first time that there is a multifaceted crosstalk between CaMKII and PKA-dependent signaling which orchestrates the overall distribution of HDAC4 between the myocyte nucleus and the cytoplasm in quiescent and electrically stimulated cardiomyocytes (Fig. 9). At baseline, the nuclear-to-cytosolic ratio of HDAC4 levels is slightly above 1.0, is limited by baseline CaMKII activity and further net nuclear export occurs upon CaMKII activation. On the other hand, β-adrenergic stimulation enhances PKA activity resulting in nuclear HDAC4 accumulation that requires phosphorylation sites S265/266. Under β-adrenergic activation, PKA has a more dominant early effect driving HDAC4 to the nucleus, while prolonged stress leads to greater CaMKII-dependent nuclear export of HDAC4. This orchestrated co-regulation between PKA- and CaMKII-dependent effects is shifted in failing cardiomyocytes, where we observed increased baseline CaMKII activity and diminished PKA response to β-AR activation. This novel finding sheds light on crosstalk between CaMKII and PKA-dependent signaling, relevant to the treatment of hypertrophy and heart failure.

Our initial experiments reveal that at rest the F_Nuc_/F_Cyto_ balance of HDAC4 is largely determined by basal CaMKII activity. Either acute inhibition of CaMKII or genetic deletion of CaMKIIδ (Fig. 1) increased resting nuclear HDAC4 levels. This baseline CaMKII functional effect was somewhat surprising, because both mathematical models [58] and experimental evidence [22] indicate that cytosolic CaMKII activity is quite low under quiescent conditions. A likely explanation involves that fact that CaMKII is docked to HDAC4 in the region of R601 [4], and their interaction may facilitate very local CaMKII activity on HDAC4, even at diastolic [Ca^2+^]. Indeed, ablation of this interaction by R601F-HDAC4 was sufficient to mimic CaMKII inhibition or knockout with respect to resting F_Nuc_/F_Cyto_. The demonstrated Ca^2+^-, CaM-, frequency- and CaMKII-dependence of HDAC4 translocation (Fig. 2) highlights the potential implication in the hypertrophic remodeling [11] in which Ca^2+^ handling and CaMKII activity [9] are both altered. The observed negligible effects of a PKD/PKC inhibitor Gö6976 on HDAC4 localization may be explained by the association of CaMKII with HDAC4 [4] and the dramatic decrease of PKD levels in the mammalian heart during development from neonatal to adult myocardium [32]. Notably, our prior work showed that in adult ventricular myocytes HDAC5 nuclear export in response to Gq-coupled receptors was roughly equally dependent on CaMKII and PKD [9, 69].

Sympathetic β-AR stimulation is a rapidly recruited mechanism to increase cardiac inotropy, heart rate and lusitropy as the fight-or-flight response. Kinases downstream of nuclear β-ARs modulate many systems in heart, including gene expression [60] but PKA effect on HDACs have not been deeply explored. Here we demonstrate that the HDAC4 nuclear accumulation seen under β-AR (Iso) or adenylate cyclase (forskolin) stimulation is due to PKA activation, which reduces the HDAC4 binding to the chaperone 14–3-3 and consequent inhibition of nuclear export as well as enhancing nuclear import. In addition, we could demonstrate that S265/266 is essential for PKA-mediated regulation (Figs. 3, 4).

Backs and colleagues showed that PKA could also bind to HDAC4 at a site near the CaMKII binding site, and that this PKA can trigger cleavage of HDAC4 at Y201 [5]. They further found that the small N-terminal fragment (containing the MEF2 binding domain, but not the HDAC domain) translocates to the nucleus and by itself inhibits MEF2-dependent transcription. This pathway could complement the PKA-dependent nuclear localization of HDAC4 we describe here. However, it cannot explain our results, which use HDAC4 with GFP fused to the C-terminus, and the HDAC4 antibody used for ICC recognizes a specific epitope at amino acids 530–631. Thus, we are not monitoring an N-terminal part of HDAC4. In addition, the S265/266 PKA target site that we found to be required for PKA-dependent nuclear translocation is not on the N-terminal fragment. So, there are likely two mechanisms by which PKA promotes elevated nuclear HDAC4-dependent suppression of MEF2-dependent transcription.

Because of the dramatic effects of S265/266A mutation on the responsiveness to cAMP signaling (Figs. 3c, 6d) it is tempting to speculate that this is a direct PKA phosphorylation site which interferes with 14–3-3 binding. Similar to our results in adult ventricular myocytes, Walkinshaw et al. [62] found a GFP-S266A HDAC4 mutant to have no basal effect on Nuc/Cyto distribution (vs WT HDAC4), but prevented nuclear localization induced by PKA overexpression in HEK293 cells or 8-Br-cAMP treatment in C2C12 cells. However, when they assessed HDAC4 phosphorylation at S266 by a custom-developed phospho-specific antibody, they found a high baseline level that was reduced, rather than increase, upon 8-Br-cAMP or forskolin treatment. Those findings agree with HDAC5 studies of a site (S279) that is analogous to S266 in HDAC4, showing that PKA stimulation caused decreased phosphorylation of HDAC5 S279 that correlated with nuclear import of HDAC5 [33, 67]. He et al*.* [33] suggested that the PKA-induced HDAC5 nuclear import was mediated by PKA-induced PKD inhibition, where PKD-dependent phosphorylation at different sites (S259/S498) is known to drive nuclear export. We cannot rule out an analogous situation for PKA effects on HDAC4 translocation. However, CaMKII has more prominent effects than PKD on HDAC4 vs. HDAC5, and we found that PKD inhibition did not promote baseline HDAC4 nuclear import (Fig. 1b) and substitution of the S265/S266 site with alanine rendered HDAC4 unresponsive to cAMP treatment (Fig. 3c). Thus, the PKD inhibition mechanism described for HDAC5 does not readily explain our PKA-dependent HDAC4 effects. However, we did find that the broad kinase inhibitor staurosporine raised basal HDAC4 nuclear levels (Fig. 1b), so some basal kinase activity, other than CaMKII, PKA, PKD or PKC, might additionally modulate nuclear shuttling of HDAC4. In conclusion, we find that S265/S266 sites on HDAC4 are required for PKA-dependent HDAC4 nuclear import, but the idea that PKA phosphorylation mediates this effect is likely an oversimplification.

Since both CaMKII and PKA are activated by β-AR stimulation [25] and they produce opposite effects on HDAC4 nuclear shuttling, we hypothesized that one might play a dominant role in dictating F_Nuc_/F_Cyto_ of HDAC4 under β-AR activation. Separately, PKA activation alone without Ca transients produced strong nuclear HDAC4 accumulation reliant on S265/266 sites, while in contrast CaMKII activation without PKA activation caused robust nuclear HDAC4 depletion, attributed to S467 and S632 phosphorylation by CaMKII [4] allowing 14-3-3 binding that drives CRM1-dependent nuclear export. When both PKA and CaMKII are activated, neither is totally dominant (Fig. 5). In our most physiological setting, where pacing rate is abruptly increased along with β-AR activation, PKA-dependent nuclear accumulation of HDAC4 predominates early during the initial response, after which CaMKII-dependent HDAC4 export is initiated and gradually drives HDAC4 out of the nucleus. The reason for this “delayed” response of the CaMKII-dependent effect may be because—in contrast to PKA—CaMKII can accumulate in the active state because of progressive increase in the autonomously active state, a kind of molecular memory [1]. Moreover, β-AR stimulation can desensitize, and there is precedent for early β-AR effects being primarily PKA-dependent while longer term effects being more CaMKII-dependent [66]. In this way myocytes may distinguish between short-term fight-or-flight inotropy and chronotropy, vs. the need for transcriptional remodeling to deal with chronic stress. That is, the short-term β-AR effect may keep or promote HDAC4 nuclear retention to prevent acute transcriptional remodeling, to focus on acute fight-or-flight PKA effects. If the stress becomes more chronic, the CaMKII pathway may progressively encourage HDAC4 nuclear export and de-repression of MEF2 transcriptional activation [24, 31, 51, 66]. Indeed, activated CaMKII also translocates to the nucleus, especially at high pacing frequencies and as hypertrophic and HF remodeling progress [41]. Moreover, simply overexpressing CaMKII in cardiac myocytes (as occurs in HF) is already sufficient to drive nuclear HDAC4 depletion (Fig. 5).

Our data from two experimental heart failure models confirmed that the balance between PKA- and CaMKII-dependent effects is shifted in diseased cardiomyocytes (Fig. 7), with baseline HDAC4 being more cytoplasmic compared to healthy controls and PKA-mediated effects being almost completely abolished in both quiescent and electrically stimulated cells. This translates functionally to a loss of the time window in which Iso stimulation can prevent the onset of HDAC4 net nuclear export upon increased workload demand in failing cardiomyocytes, presumably further worsening the remodeling process.

Notably, the temporal situation reported here for HDAC4 with respect to PKA-dependent nuclear retention and CaMKII-dependent nuclear depletion is analogous to what we found earlier with HDAC5 [12], although the phosphorylation sites differ and HDAC5 depends at least as strongly on PKD as on CaMKII. Thus, this may be a generalized characteristic of multiple Class II HDACs, to distinguish between acute fight-or-flight effects and activation of transcriptional adaptation.

We also found here that non-failing human ventricular myocytes exhibit quite similar CaMKII and PKA-dependent HDAC4 translocation as seen in rabbits, although limited tissue access preclude full analysis of the crosstalk in human myocytes. Nevertheless, it seems this same molecular pathway may well occur in human hearts. Both β-AR blocking agents [15, 52] and/or angiotensin-converting enzyme (ACE) inhibitors [16, 55] significantly improve survival rates for patients with chronic heart failure. The fact that Iso and Ang II-dependent CaMKII activation shifts HDAC4 in opposite directions, but downregulation of β-AR in HF coupled to upregulation of CaMKII signaling in heart failure may limit the MEF2 repressive effect of PKA and allow the CaMKII-dependent nuclear export to become more dominant. Indeed, our experiments in cardiomyocytes from failing human hearts showed both chronic CaMKII-mediated HDCA4 nuclear export and diminished PKA-mediated nuclear accumulation (Fig. 8). Thus, the combination of β-AR blockers that limit functional downregulation of β-AR and inhibition of Ang II-dependent CaMKII signaling may combine to limit HDAC4 nuclear export and keep MEF2 repressed.

In summary, in adult ventricular cardiomyocytes, CaMKII plays a significant role in determining the localization of HDAC4 in quiescent cells and during Ang II stimulation or under high frequency pacing. In contrast, β-AR stimulation leads to PKA-mediated nuclear translocation and retention of HDAC4 that depends on the presence of regulation of S265/S266 on HDAC4. When both kinases are activated simultaneously, PKA plays an initially dominant role, but gives way to longer-term CaMKII-dependent nuclear export of HDAC4. This tightly regulated balance between PKA- and CaMKII-dependent effects in disturbed in diseased cardiomyocytes, where CaMKII-mediated export is enhanced and PKA-dependent nuclear accumulation is diminished. Importantly, our results extrapolate to human ventricular myocytes, underscoring the clinical importance of PKA-CaMKII crosstalk for hypertrophy and heart failure patients.

## Supplementary Information

Below is the link to the electronic supplementary material.

Supplementary file1 (DOC 308 KB)

## Data Availability

The data supporting findings of this study are available from the corresponding author upon reasonable request.
