# Supplementary material for: CaMKII and PKA-dependent phosphorylation co-regulate nuclear localization of HDAC4 in adult cardiomyocytes
Source: Basic Res Cardiol. 2021 Feb 15;116(1):11. doi: 10.1007/s00395-021-00850-2 (PMC7884572; doi:10.1007/s00395-021-00850-2)

# Electronic Supplementary Material

**Supplemental Fig. 1 *CaM levels in isolated ventricular cardiomyocytes***

**(a)** A representative Western blot using an antibody against calmodulin (CaM) compares freshly isolated myocytes (lane 1) to cultured myocytes overexpressing CaM (lane 2) and to cultured myocytes with no CaM overexpression (lane 3). Note how addition of AdV CaM prevented the decline in CaM expression seen during the normal culture process. **(b)** A Coomassie stained gel was used as the loading control for the 3 lanes.


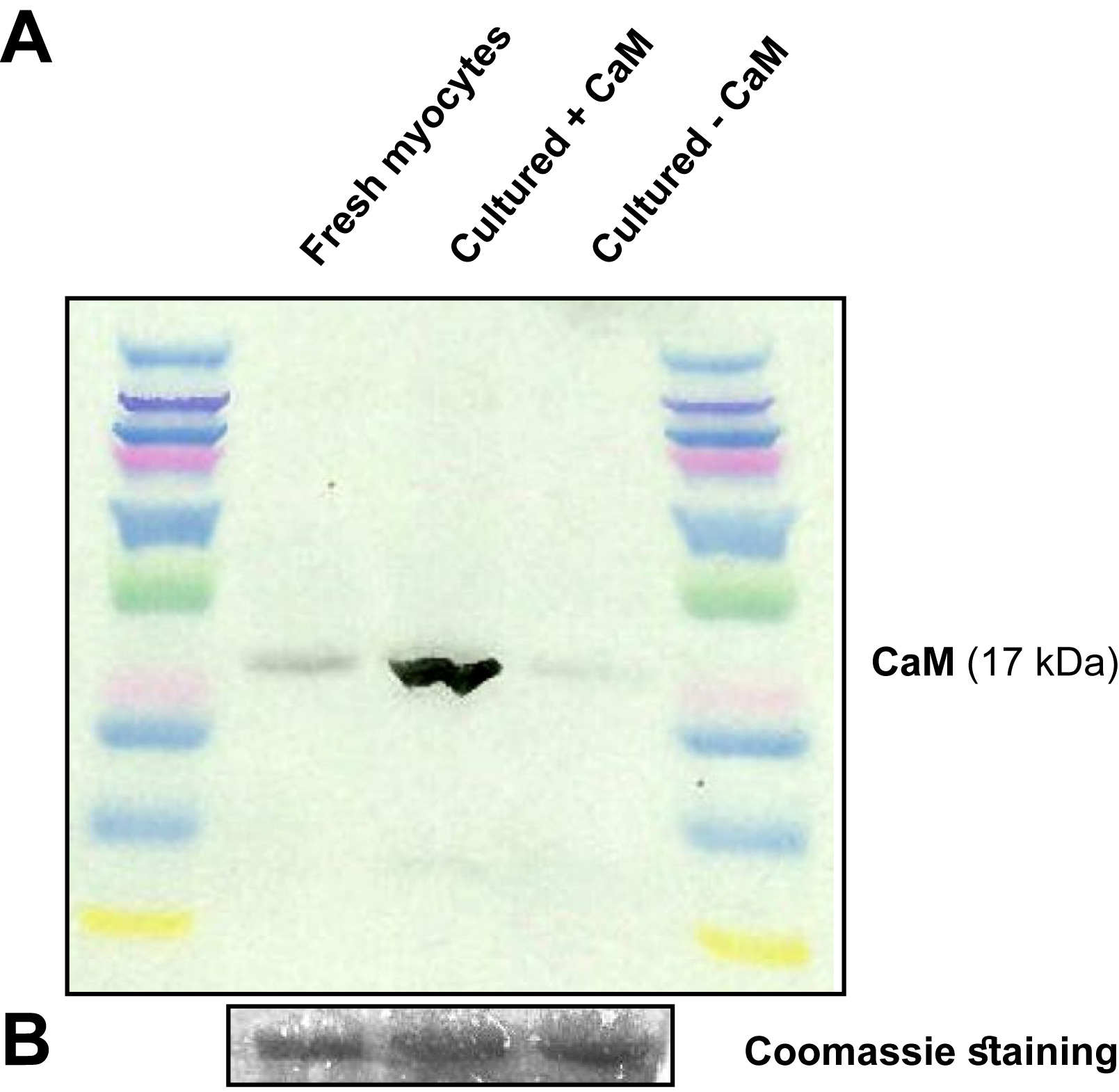

Supplement: Supplementary file 1 — Supplementary file1 (DOC 308 KB) [file 395_2021_850_MOESM1_ESM.doc]
